# Gambling in the Landscape of Adversity in Youth: Reflections from Men Who Live with Poverty and Homelessness

**DOI:** 10.3390/ijerph13090854

**Published:** 2016-08-31

**Authors:** Sarah Hamilton-Wright, Julia Woodhall-Melnik, Sara J. T. Guilcher, Andrée Schuler, Aklilu Wendaferew, Stephen W. Hwang, Flora I. Matheson

**Affiliations:** 1 Centre for Urban Health Solutions, Li Ka Shing Knowledge Institute, St. Michael’s Hospital, 30 Bond St., Toronto, ON M5B 1W8, Canada; woodhallmelj@smh.ca (J.W.-M.); sara.guilcher@utoronto.ca (S.J.T.G.); schulera@smh.ca (A.S.); hwangs@smh.ca (S.W.H.); mathesonf@smh.ca (F.I.M.); 2 Department of Health, Aging, and Society, McMaster University, 1280 Main St. W., Hamilton, ON L8S 4M4, Canada; 3 Leslie Dan Faculty of Pharmacy, University of Toronto, 144 College St., Toronto, ON M5S 3M2, Canada; 4 Institute for Clinical Evaluative Sciences, 2075 Bayview Avenue, Toronto, ON M4N 3M5, Canada; 5 Good Shepherd Ministries, 412 Queen St E., Toronto, ON M5A 1T3, Canada; aklilu@goodshepherd.ca; 6 Dalla Lana School of Public Health, University of Toronto, 155 College St, Toronto, ON M5T 3M7, Canada

**Keywords:** youth, problem gambling, complex needs, trauma, qualitative, homelessness, case study

## Abstract

Most of the research on gambling behaviour among youth has been quantitative and focused on measuring prevalence. As a result, little is known about the contextual experiences of youth gambling, particularly among those most vulnerable. In this paper, we explore the previous experiences of youth gambling in a sample of adult men experiencing housing instability and problem gambling. We present findings from a qualitative study on problem gambling and housing instability conducted in Toronto, Canada. Thirty men with histories of problem or pathological gambling and housing instability or homelessness were interviewed. Two thirds of these men reported that they began gambling in youth. Five representative cases were selected and the main themes discussed. We found that gambling began in early life while the men, as youth, were also experiencing adversity (e.g., physical, emotional and/or sexual abuse, neglect, housing instability, homelessness, substance addiction and poverty). Men reported they had access to gambling activity through their family and wider networks of school, community and the streets. Gambling provided a way to gain acceptance, escape from emotional pain, and/or earn money. For these men problematic gambling behaviour that began in youth, continued into adulthood.

## 1. Introduction

Youth gambling is acknowledged as an important health issue [1,2]. Historically, youth gambling has received less attention in the academic literature than adult gambling and other adolescent problematic behaviours, such as drug and alcohol use [1], yet there is a growing recognition of the prevalence and severity of problem gambling among young people (see Volberg et al. [3] for a review). Prevalence rates of at risk, problem and pathological gambling among adolescents are higher than in adults in North American, European and Nordic countries [3,4,5,6,7].

General population studies suggest that prevalence rates for problem gambling among youth aged 18–24 range from 2% to 8%. [8]. Recent studies found that rates of problem and/or pathological gambling among adults experiencing complex vulnerabilities such as homelessness and poverty, are much higher in adults than the general population, up to as high as 35% for pathological and problem gambling combined [9,10]. To date, one study has measured problem gambling among a Canadian homeless youth cohort and found that 12.6% of youth surveyed exhibited at risk gambling behaviour [11]. This estimate suggests a much higher prevalence of problem gambling among vulnerable youth than in the general population, highlighting the need for greater understanding of the contributing factors.

There are several known risk factors for gambling in youth which include early life exposure to gambling, initiation into gambling by family members and poor family cohesion [4,12]. Gambling is associated with increased rates of alcohol use, drug use/dependence, depression, anxiety, and other psychiatric problems, as well as lower self-esteem among youth [5,13,14,15,16]. Youth who gamble experience poor school performance, financial strife, disrupted family relationships, and have higher rates of delinquent behaviours [1,8,11,17]. Adolescents gamble for a variety of reasons, among the most common, according to Kristiansen and Jensen [18], being to win money, for fun, to socialize with friends, and to alleviate boredom. The authors also found that youth who were at-risk gamblers were more likely to report gambling to escape problems, or because of an inability to resist temptation [18]. Male youth, who have a tendency towards risky behaviour (e.g., substance use and delinquency) are particularly vulnerable to problem gambling [19,20,21,22].

Those who gamble at a younger age, and gamble excessively, are considered to be more susceptible to developing gambling problems as adults [23,24]. Delfabbro, King and Griffiths [23] found that youth gambling patterns varied considerably from year to year, but that particular risk factors are associated with persistent gambling in youth and into adulthood. Youth who had an earlier initiation into gambling and had experienced a big win when they began gambling were at particular risk of adult problem gambling [23,25]. Yet, little is known about social contexts of early gambling experiences [26] and whether youth gambling persists into adulthood [1].

Storr et al. [27] and Lee et al. [28] reported that youth who participated in gambling activities experienced more adverse life events such as threats of harm, violence or deviant activity. Research shows that there is an association between childhood experiences of trauma and adversity and adult problem gambling [29,30,31,32]. A case study by Lee, Solowoniuk and Fong [30] found that both substance use and childhood adversity (including childhood maltreatment) were associated with problem gambling among male and female adults. More recently, Saugeres et al. [33] found that childhood experiences of family dysfunction and gambling in the family contributed to adult problematic gambling. Scherrer, Xian, Kapp, Waterman, Shah, Volberg and Eisen [29] also found that childhood and lifetime traumatic events (e.g., child abuse, child neglect, physical attack) were significantly associated with pathological gambling in adulthood [29]. However, they were unable to determine whether early life trauma contributed to the onset of gambling in youth. Thus, there is a gap to fill in the understanding of the connection between trauma and youth and adult gambling behaviour.

Existing literature on youth gambling tends to be quantitative, focusing mainly on prevalence rates [1]. There is an identified need for qualitative inquiry in the field of adolescent gambling, including an understanding of issues of comorbid gambling and other addictive behaviours [1], the “antecendents of problem gambling among youth” [34]. Reith and Dobbie [35] argued for a greater understanding of the deeper meanings and contexts of early gambling behaviour. There is emerging evidence that gambling in youth may be predictive of problem gambling in adulthood but findings have been contradictory [23,36]. Moreover, the experiences of youth that lead to early gambling and problem gambling behaviour later in life are not adequately understood [13]. While there is a small but growing body of research on problem gambling and homelessness, we only found a single study that examined gambling behaviour among youth experiencing housing instability and homelessness [11]. Dufour, Roy, Boivin, Boudreau and Robert [11] concluded that “certain” homeless youth might have problem gambling, but that further exploration is needed to identify the relationship between problem gambling and other at risk behaviours among youth (e.g., substance use). These gaps in the literature suggest that we need a more in-depth understanding of the social and material landscape of youth gambling, especially among those experiencing marginalization, or vulnerable circumstances. The aim of this paper is to explore youth experiences of gambling behaviour through the reflections of men who experienced problem gambling in adulthood and who were clients of a community-based agency which provides a range of services for homeless and marginally housed clients in a large urban centre.

## 2. Materials and Methods

This research was conducted in collaboration with the Good Shepherd Ministries (GSM) of Toronto. GSM is a community-based non-profit organization that provides housing, health, and social services for homeless and marginally housed men. This study received ethical approval from the Research Ethics Board of St. Michael’s Hospital (project identification code: 13-180), Toronto, Canada, and was conducted in accordance with the Tri Council Policy Statement: Ethical Conduct for Research Involving Humans. The findings in this paper are drawn from the second stage of a two stage research project. The first stage, also conducted in collaboration with the GSM of Toronto, assessed the prevalence of gambling among their clients, with 264 clients screened for problem gambling between 4 March and 15 May 2014 using the National Opinion Research Centre Diagnostic Statistical Manual (DSM) Screen for Gambling Problems (NODS) [37] and its sister short screener, the National Opinion Research Centre Diagnostic Statistical Manual (DSM) Screen for Gambling Problems—Control, Lying and Preoccupation (NODS-CLiP) [38]. The NODS screening indicated that a large number of clients were at risk of lifetime (8.3%), problem (9.5%) and pathological gambling (24.6%) [9]. Scores on the NODS correspond to gambling status as follows: 0 indicates non-problematic gambling; 1 to 2 indicates problematic gambling; 5 or higher indicates a likely diagnosis of pathological gambling, which is consistent with the diagnostic criteria of the Diagnostic Statistical Manual of Mental Disorders, 4th Edition (DSM-IV) [39]. The section below discusses the methods used for the follow-up qualitative study which explored gambling and housing instability in a sample of men who participated in the prevalence study.

The clients who participated in the prevalence study were asked if they would be willing to provide their contact information for a future study about gambling. For those who agreed, we collected personal contact information in the form of first and last name, date of birth, phone number and email address. They were also asked to provide contact information for any family members, service providers, friends, and GSM staff who would know their whereabouts should their contact information change. Eighty two men agreed to be contacted. We randomized the list of men by name and selected participants sequentially to be contacted. Of the 30 participants who were located and agreed to participate, four were recruited via email and the rest were recruited by phone. The sample size is appropriate given the aim of the study was on phenomenal experience. For example, Morse [40] recommends that ethnographic and grounded theory studies comprise between 30 and 50 interviews.

We hired peer interviewers (people with lived experience of mental illness, addictions, and homelessness) to conduct the interviews. These peer interviewers were provided with intensive training on the project background, qualitative and face-to-face interviewing skills, self-disclosure, safety, and principles of rigor in qualitative research. A more detailed discussion of our engagement of peer interviewers is available elsewhere [41].

Five pilot interviews were conducted in October 2014. An additional 25 interviews were conducted between November 2013 and February 2014. Participants completed a single interview which ranged from 30 to 90 min in length.

Each participant was asked to provide demographic information. Gambling activity was assessed using the Canadian Problem Gambling Severity Index, distress was assessed with the K10 [42], and drug and alcohol use were measured using the WHO ASSIST [43]. We developed a semi-structured qualitative interview to elicit men’s perceptions and experiences with gambling, housing, and access to support services. Participants received a $50 honorarium for participation. Due to the sensitive nature of the information being captured, we required verbal consent for the interview and for the audio recording of the interview. All participants gave their consent to be interviewed. Interviews were audio recorded and transcribed verbatim. Participants were assigned pseudonyms that reflected the men’s age and ethnicity, while protecting their identity.

Grounded theory guided our data collection and analysis [44]. The coding followed Burnard’s proposed method [45]. Open coding was used to analyze seven transcripts. These seven interviews were selected to represent different points in the data collection process (1 pilot and 6 post-pilot), data collection by each of the peer interviewers, and different participant scores on the Canadian Problem Gambling Severity Index (CPGI). After reviewing the assigned transcripts, the team met to discuss the initial codes and to develop theme names and descriptions. We then conducted focused coding wherein each team member independently coded a selection of 11 transcripts, including the seven previously used in open coding. As new codes emerged they were integrated with the initial codes list and these formed the basis for coding the full set of 30 interviews. The study peer interviewers assisted with validation of the themes. In total, 64 nodes were developed by the coding team to represent the themes that emerged in the data. All coding was performed using NVivo 9.2 (QSR International Pty Ltd., Melbourne, Victoria, Australia). 

Youth experiences represented one of the initial nodes identified by the research team. This node provided the data for the current analysis as it captured men’s experiences with gambling, family, adversity, and housing. The men’s reflections on youth gambling was an emergent finding, not the main goal of the larger study; as such we chose to include a broader range of ages for youth. Two thirds of the men in the sample (*n* = 20) indicated that they began gambling in early life (anywhere from the age 6 to 22) and also described experiences of adversity in youth. This group of men constituted the subsample for the present analysis. Data from these 20 men were further explored through a comprehensive query process in NVivo that involved searching for overlaps between youth experiences and other salient themes in the study such as, substance use and abuse, relationships, criminality, and reasons for gambling. The five cases presented below were chosen because they represented depth of data on the emergent themes described by the larger sample and because they best exemplified the combination of factors that represented complex vulnerability in men’s lives as youth and the reasons they turned to gambling as a coping mechanism. These themes included beginning gambling before age 22, experiencing gambling in conjunction with substance use, homelessness/precarious housing, introduction to gambling by peers and or family members, and relying on gambling to cope with trauma, poverty or low self-esteem.

In presenting these five cases, we highlight landscapes of youth adversity and describe the social relationships that first exposed them to gambling behaviour. We also discuss the meanings that these men found in gambling, as our data illustrate that the men formed meaningful relationships with gambling that extended from youth to adulthood.

## 3. Results

In analyzing the detailed descriptions men provided about themselves, their lives, and the reasons they began gambling, we found that youth was a critical time period in which the men began gambling. The landscapes of these men’s early lives were marked with trauma, poverty, family violence, and neglect, which we describe using the term complex vulnerabilities (see Table 1). Many were exposed to gambling through social influences such as family and peers. Once gambling was learned and experienced within settings of deprivation, it became an activity that provided enjoyment and helped them cope with difficult circumstances of trauma, poverty and homelessness (see Table 2). For many men in the study, a meaningful relationship with gambling began in youth and extended through their adult lives. Using a case study approach, we present the narratives of five men whose descriptions of gambling and youth resembled those told by other men in the study.

Mark

Mark was born and grew up in Canada. He described his early childhood as “rough” and disclosed that more than one family member sexually assaulted him repeatedly as a young boy. He struggled with severe Post Traumatic Stress Disorder and insomnia. He explained that both of his parents abused alcohol and he has memories of family violence.

My parents were alcoholic. So, very heavy alcoholics, always fighting, arguing. We would get beat, our Christmas tree, we would go out the door because they were so drunk. They’d throw it out, but there was never birthdays. You could never take Valentine’s into your friends at school. We never had an Easter. I remember one time it was my birthday, and my dad took my cake and just threw it against the wall, and then, ever since then I’ve never celebrated birthdays.

Mark experienced housing instability throughout his childhood, moving multiple times in Ontario which interrupted his education. In school, Mark felt singled out and “bullied,” which is a common experience expressed by other men who began gambling at a young age. Mark expressed how this had negative impacts on his self-esteem.

I’ve always moved around like in different areas, all over the place. Because my parents, when they were drunk, didn’t pay the rent and that, and so, I lived a rough life as a kid…anywhere we lived, and it really screwed my life up. I went to many, multiple schools. Because my parents were heavy duty alcoholics, I would have to go to school in my dirty, raggedy clothes, and the kids would make fun of me. I think that’s, like I have a very low self-esteem.

In order to cope with the pain of abuse and disruption in his early life, Mark began to abuse alcohol at seven years old. This coincided with the first time he was sexually abused. Despite attempts by authorities to keep the family together, Mark left home at eighteen years old. These early experiences set Mark on a life trajectory of instability often living on the run.

I started drinking when I was 7 years old...I’d drink to get numb, and just to hear the steps if somebody was coming to hurt me. Then when I was 18, I looked at both my parents....numerous times I called the police, and back then, the police and the Children’s Aid wanted to keep the family together, they didn’t want to separate the family. So, they tried working with my parents and that, and then, when I was 18 I looked at my parents and I said, I’m out of here, and because it (sexual assault) started when I was like 7, I was afraid to sleep. I developed insomnia.

Mark started gambling at fifteen , which was facilitated by his older sister. Mark’s example of being exposed to gambling by a family member (e.g., parents, uncles, and siblings) is similar to several other men in the study. Mark began by helping his older sister scratch lottery tickets. As he began to gamble himself, he enlisted his sister’s help to purchase lottery tickets for his own use.

I used to get my older sister to go in and get me, I don’t know if you remember them, the Wintario. I used to get her to get me the Wintario tickets. If I won on it, my sister would cash it in for me. …My sister used to stay out and buy me that, and I used to scratch the ticket for her.

Mark’s sister modeled early gambling behaviour for Mark through her purchases of scratch tickets and also through her participation in Bingo. His sister’s experience with gambling was influential to the development of his own gambling addiction. When asked why he began gambling, Mark said that he observed his sister’s behaviour and her reaction to winnings.

When I see my sister, she used to go to bingo. She used to come home and say, “Hey I won, I won”. She used to win $500, $1,500.

When Mark experienced the feeling of winning himself, he poignantly remembered the moment he became addicted to the feeling gambling gave him.

To see the rush, and all of that excitement. Yeah. Like even, I remember when Wintario brought out the scratch on the bottom of the ticket, and you could win anywhere from $5 to $100,000... and the first time I ever won $200 on that ticket, like I almost tore the guy’s store apart. It was the adrenalin I had. It was like, wow. I finally won, and ever since then, and I was 15 years old, since then, I got that adrenalin.

At fifteen years old, Mark experienced his first gambling related adrenalin high and has been gambling ever since. Gambling remained interrelated with alcohol and drug use for Mark throughout his life, as was a common theme for most men we interviewed. As an adult, he described how he still turns to gambling to “ease his mind” as he still suffers from Post-Traumatic Stress Disorder. Mark is in treatment for substance addiction, but doubts that treatment for his gambling addiction would help him.

Gambling. As I said, it’s my... it eases my mind. I don’t even think if I went to a gambling, if there was one out there, a gambling detox, whatever the hell you call it. I don’t think it would help me. Because, I get that urge. I get those nightmares. I wake up in the morning and oh, I want a scratch ticket now.

Steve

Steve was born in a Caribbean country and immigrated to Canada as a young adult. He was born to very young parents and experienced complex vulnerability in his early life. Like Mark, he recalled being physically abused, exposed to alcohol consumption by his father in early childhood, and lived in constant fear. Steve first ran away from home at eleven years old to escape his father’s abusive behaviour.

When I was born my mother and my father were 14 years old. The first time I tasted alcohol, I was four years old. My father give me that drink, and he gave me that drink just to see how I would react, and ever since that first drink, anytime I get a chance to drink, I will drink. At age 11, I had to run away from home because my father was very abusive, and I was afraid of him. He used to physical abuse me for everything I have done wrong.

He was taken by an aunt to live with his mother, but that proved difficult because be developed a drinking problem, got involved with petty crime, and his behaviour was unmanageable for her. He found himself back on the street at age twelve within a short period of time.

I was living with my mother for a while, and there, I started drinking a lot, and I started to hang with a group of guys who were much older than me and they used to give me a lot of alcohol to drink, and they used encourage me to do crime. Like, petty crime, like going into people’s back yards, steal their bottle, steal their gas, and so forth, and my mother didn’t like that, and I had to leave from my mother’s home.

Steve was exposed to gambling on the streets by the “crooks and criminals” in his social network. He felt pressured to use gambling as a way to survive on the streets. Steve described how he had little choice but gamble to earn recognition of the “older professional crooks”. He described below that he “learned” how to gamble, by watching the game unfold and being an “assistant” or as he described a “poonta” to the “bigger” guys. He played the games he needed to play to “hang” with the gang; in essence this older gambling crowd became his social network.

Well, I think what led me to gambling was the kind of people that I was around, right, because they were like older, professional crooks, gamblers all they want to do is... they live to gamble and gamble to live. They gamble all their life. They were like criminals, and I was in a position where I had no choice but to join them because I was homeless, and there I start hanging with them. When I was about 12 years old I was living on the streets, and there was a few bigger guys that I used to hang around, and they were gamblers. They used to play a game called Crown Anchor where they have six or three dice underneath a cup, and they have a corrupter under the board. …So, he would have a hoard of people around and I would be at the side …I would be there betting as if I’m betting for real to entice people to come and bet. If he wins I would get a little small amount, but he pays me, I would use that money to play cards. As I get a little bit more experience about the game what I used to do is I used to have one guy who’s playing in the game who him and I are partner, and we used to know what the 52 card in the pack means by sign …So, whatever he wants, I would let go out of my hand, you know, and stuff like that, and he would shuffle the deck in a way we know how to cut the deck for him to wins.

He did not like the way he was treated by the gambling “professionals”, on the street, as he called them. He continued despite this mistreatment. He described how he eventually developed enough gambling skills that he felt he was able to go out on his own and support himself.

So, there was two guys in my community that they used to use me …so that people could bet, and when they win big sum of money, they would give me like a small amount and they would take the bigger bread. But I, as a little kid, didn’t really like it because I know they were treating me (badly), but I couldn’t do nothing about it because they were bigger, stronger guys than me, and one day, I tried to play the game, and the result of I try to play the game, because they were professional game, they are the man. They have the money. They are playing the game, right, and I started to play the game really good, and I become a pro in the game. I said, “I don’t want to work with you guys no more. I’m on my own”. So, here I am all by myself in the marketplace playing the game.

Steve did not want to return to his father, for fear of his abusive behaviour or his mother, because she could not cope with his behaviour. Having no other means to earn money, he felt unable to escape from this lifestyle. Gambling was a double-edged sword in his life. Gambling gave him a “buzz” similar to an adrenalin rush, a high not unlike drugs. Eventually, his gambling behaviour became a detriment, exhausting his funds to buy even basic necessities.

I did this for years, and every single day, I remember going to the gambling house. I would leave that gambling house with not a penny in my pocket. Hungry, have no place to live, my breath it stinks. I haven’t showered because I used the money to buy soap, to gamble and I lost all my money. The next day, I get up, that’s what I want to do again, and it’s like I used to get high. I used to get a buzz off of having money, and sitting around the gambling table. Yeah. And I did it for years.

For Steve, gambling was a skill and self-perceived means to survive poverty and homelessness. Gambling also provided him with a social circle. Being with others involved in the gambling scene (his network or community) satisfied a need in his young life. Having money in hand was a high in and of itself. He also felt that gambling helped him survive poverty—“most of my life, gambling, I was in need.”

Fahad

Fahad immigrated to Canada from an African country when he was five years old. In early life, like Mark and Steve, Fahad experienced physical abuse, housing instability, and family violence. Fahad’s father was abusive and he witnessed his father’s pathological gambling and alcohol consumption. His father’s gambling addiction led to the loss of their family home.

I had a really, I guess you could say, a traumatic childhood because of my dad. He was an alcoholic, an abusive alcoholic, and I always thought I would never be like my dad, but unfortunately, as time progressed, I eventually became an alcoholic myself …I guess you see in my past, my parents, we ended up losing our house too because my dad ended up not only drinking but he also gambled the house away that we lost.

Fahad began drinking and gambling in high school. His experiences were similar to those of other men in the study who began gambling and drinking in the social environment of school. Fahad, a self-confessed “football fanatic” began gambling by betting on sporting events, a theme re-iterated by several men in our study. Gambling became a regular activity for Fahad and developed into an addictive and “vicious cycle” reinforced by winning “a lot” of money.

I started gambling when I started drinking. So, that would be at the age of 16, and I went on with drinking, I went on with gambling... Like I’d gamble on a daily basis when... I used to like ProLine, on sporting events like basketball, or hockey, baseball, soccer, football. I’m a football fanatic. There was one time when I won a lot of money in ProLine, and it was like $2,000, and I took the $2,000 and I spent it all over again on gambling even more money. Like all of it just so I could win more. I guess you could say it’s a vicious cycle like it’s never enough. I mean you think $2,000 is a lot, yes it is, but when you could win more, why not spend more?

As a minor, Fahad developed strategies to persuade adults to gamble for him. He does not specify the relationship of these older individuals in the interview, so it is unclear if they are family members, or people that he connected with through gambling venues. He would also bribe the people that he drank with.

When I was that age, I couldn’t gamble, or drink, so I had to pay someone to play for me. So, they’d have to get something out of it. Say if you want me to do it, “what’s in it for me?”. So, I’d have to give them some money so they’d play for me because you ain’t going to get something for free that’s for sure. “What’s in it for me?”, they’d keep saying that. “Okay, I’ll give you an extra $20 just go play for me.”... but most of these people have been screwed over a lot of times that I’d give them the money up front first, and I wouldn’t see them. So, I’d have to get like my people that I drank with to buy the tickets because then I’d bribe them by buying alcohol. That would work. Instead of giving them the money because I’d spend the money on a drink. So, I’d say, okay. I’ll buy you an extra beer. I’ll buy you the first two rounds, and because they were older than me they would do that. They would play for me because they’d get something out of it.

Fahad continued gambling because it enabled him to make money, a theme we heard Steve and others describe. He used this money to sustain his alcohol addiction. He enjoyed the process of bribing others and saw this as a skill. For Fahad, like other men in the study, gambling also provided hope. For him, this meant the chance that gambling could provide him with a better lifestyle. He described these interrelated reasons for continued gambling in his youth.

So, the reasons for gambling would be not only to win more money but at the same time I’d be able to drink more. Besides that I’d be also to bribe people, and I guess live above my needs.

Elliot

Elliot was born and raised in Canada. He shared very little about the details of his early life except that he had a father that he did not talk to and he was out on his own at sixteen. Like the other men in the case studies, Elliot needed to support early independence from his parents, supporting himself financially. He began betting on sports in adolescence, and soon after starting using substances. He reported using cocaine since he was seventeen years old.

I was betting on sports since I was 15 years old, and I been using drugs since I was 17 years old, and as a young child, sports betting for me, I was very good at it.

At the time of the study Elliot was still a very avid gambler. He was known for his gambling abilities in high school and he used these “skills” to support himself as a sixteen year old living on his own.

So, it was like, this is great. You know, so for me it was like a skill. It’s like I’m giving them my knowledge, and they’re winning money off of it, and that’s how it began for me, right. So, all through high school, I was living on my own. I moved out of my house at 16, I was on my own at 16, and that’s how I supported myself. I was able to do it, and I was very disciplined, you know, I never got into trouble or anything like that, you know, and actually high school was fun. It was really fun. So, that’s how it began for me.

Elliot described how he leveraged his interest and skill in sports to earn popularity and recognition. Similar to other men in the study, the theme of sports betting emerged as an important pathway into gambling in early life. Like Mark, Elliot acutely remembered his first time getting the thrill or rush of winning.

My first ever bet was $100, as a 15 years old high school kid, that’s a lot of money, a hundred dollars, especially in the early 90’s. I’ll never forget too, it was the New York Rangers playing the San Jose Sharks. It was a Friday night too. It was a hockey game, and the line on that game, the Rangers had to win by four goals. So, it was three and a half, because back then there was a thing called a puck line. So, in Vegas, if you’re betting the Rangers, they had to win by four goals. If you bet the Sharks, they could lose by three goals or less and you win. So, I put a hundred dollars on the Rangers, they won that game seven to one. I remember walking down (the) street and seeing the score through a restaurant window on a TV. Of course, I was happier than a pig in shit, right. So, then, and see growing up for me, I was a sports junkie. I’ve always been a sports junkie. So, I knew a lot about these games as a kid and stuff like that.

Elliot described the social reinforcement he gained from his skill at gambling in high school. His high school friends valued his gambling talents. They would ask his advice on best picks to place bets on sports teams. He described how his gambling activity led him to involvement with more “unsavoury” characters who would ask for his advice and pay him money for his picks. Similar to Steve, adult gamblers played a role in facilitating Elliot’s habit and bolstering his sense of belonging and skill.

So, for me, it was just me and my friends. They used to come to me. “Who do you like?” ProLine came out in the early 90’s right when I was going to high school. So, every day, I had everyone coming up to me. “Give me games, give me games”, because I knew the stuff inside out, I did, as a kid, that’s all I did was sports, right, even to this day. So, as I got into my later teens, 16–17 I used to frequent these places that were frequented by some unsavory characters. I guess you could say, older men who had some ties to, you know, and even these guys would come to me for games. So, I’m this kid. I’m thinking these guys over here are asking me for the game. I would give them games and the games would win, and they would give me money for it. So, it was like, this is great.

Elliot’s gambling addiction (though not recognized as such by the participant) coexisted with a drug problem in adolescence which continued into his adult life. For Elliot, gambling and drug use were interconnected. He used his gambling skills as an “avenue” to obtain drugs in high school.

Once I started doing drugs, sports betting became an avenue to obtain more drugs. See, a lot of people that I’ve come across, they have a drug addiction they got to commit crimes and all these things. I didn’t have to do that. I had this other skill that I could use in order to obtain my drugs, right.

For Elliot, his gambling “skill” and the recognition he received from it was and still is a big part of his sense of self.

It was an ego thing. Ego. So, I got these, like I told you, I got these mobster guys coming to me for picks, I’m a kid. You feel like a champ. You’re making money for these guys. It’s an ego. It makes you feel good. Absolutely.

Nolan

Nolan was a young adult at the time of the interview and his experiences represent a more current picture of youth vulnerability. He also experienced adversity in early life. His experiences were similar to many of the other older men who reflected back on their experiences of initiation into gambling in their early childhood. Nolan expressed his impression of early exposure to gambling through the individuals associated with his family’s social circle.

As a young child growing up in a situation where I was always around bikers, murderers and drug dealers in my first years of life, there was a lot of gambling going on, there was a lot of talk about gambling. There was a lot. So, as I got older, I just, you know, I started doing a little gambling myself, doing off-track betting, and hanging around drinking beers, doing off-track betting.

As an adolescent, Nolan got into trouble with the law. He was bounced around “the system” which affected his schooling. He described this as being “very hard” for him.

So, I’ve never stayed out of jail longer than three months in the last 10 years. So, from the age of 14, I was charged for the first time when I was 13, but I never went to jail until I was 14 or 15, but from that age, I never stayed out of jail longer than three months… So, you can’t go to school, you can’t graduate, you can’t even have a child in three months… So, to really grasp that, it’s kind of like... I don’t know. I mean, it’s been very hard.

Like the other men, Nolan experienced housing instability at an early age. He was “kicked out” of his house at seventeen years old as his mother could not cope with his criminal behaviour.

My mom kind of just didn’t know what to do with me. She gave up. I kept coming in and out of jail, in and out of jail, in and out of the house at 2:00 in the morning, and she met a fellow, and they were together, and they have been together for a long time now, and they thought it was the best idea if I just left the home. So, that basically... got kicked out of my home at seventeen. The same day I got out of jail, they told me, and that’s it.

At the same age he became homeless, Nolan began gambling. He noted there was “something in his history” which caused him to gamble. Like Mark and Elliot, he also expressed that he experienced a large win (for an adolescent), which influenced his desire to continue gambling. Once he was able to legally buy scratch tickets, his gambling escalated and he says he never turned back.

I guess it was just I was 17 and there was something in my history, and then, I turned 18, and I could start buying scratch tickets, and I never turned back… I just continued because I took, I got a bell, I got three bells, and I won $500, that was a lot of money for me at the time, and I just kept buying them. I just kept buying those, and kept tearing them, tearing them.

Nolan shared that, in addition to his gambling behaviour, he also had a drinking problem. He felt gambling was “natural” when he was drinking, as it seemed to lower his inhibitions. Nolan had an extensive history of delinquent behaviour, which was interrelated with his gambling activity, for example he reported that he often resorted to stealing to fund his gambling.

Nolan felt that he continued gambling in his life for “personal gain” and because it made him feel exhilarated. Gambling was, and still is, an escape that helps Nolan avoid painful memories.

It (gambling) made me feel exhilarated. I felt exhilarated even if I was taking my chances with a scratch ticket,... it was almost exciting the way it was just, I just enjoyed it so much, and was like a pasttime for me. It was like almost a hobby. So, it was something to keep my mind off other worries I was having in my life, whether it be housing, whether it would be using substances, whether it would be family issues. I just felt it was a hell of a lift. I still do.

## 4. Discussion

This paper explores men’s reflections of their gambling experiences during their youth through case studies of five participants. To our knowledge the progression of adolescent gambling is seldom reported on by adults who currently experience problem gambling and precarious housing. These individuals’ voices are often excluded from traditional research as a result of their marginalized status in society [46]. This paper therefore makes a unique contribution to the body of literature on problem gambling. The findings reveal that for adult men who live with problem gambling and homelessness, youth is a critical time period in which they begin gambling. Men’s stories illustrate that early exposure to and adoption of gambling exists within the context of familial abuse, neglect, childhood poverty, homelessness, and participation in risky behaviours such as substance misuse and criminality.

One of the central findings of the study is that gambling became a meaningful part of the lives of these men during their youth. For example, the men gambled to earn money for basic needs, for social reinforcement or to experience an adrenalin rush. Previous research asserts that youth exposure to risk (e.g., maladaptive group norms, lower neighbourhood cohesion, and familial and peer participation in maladaptive behaviours) instead of protective factors that promote resiliency (e.g., safe space, support and inclusion, and self-worth) is associated with problematic substance use [47,48]. The present study illustrates a similar relationship between gambling and resilience in youth. In the absence of protective factors (e.g., strong social bonds) gambling becomes a means for the youth to cope, to support themselves when they are forced to leave home, to enhance self-esteem and to fit in with deviant peer networks when traditional peer networks were not accessible. Our findings suggest that exposure to gambling and other risk factors such as childhood abuse, poverty, family violence, exposure to substance use and housing instability in early life may set the stage for adult problem gambling. The entrenchment of gambling as a coping mechanism in youth puts men at risk of problem gambling in adulthood. To the best of our knowledge, the present study is the first to describe men’s experiences of early adversity and traumatic events as they relate to problem gambling in youth and its persistence into adulthood.

The five case studies reveal that participants lived in environments of neglect and abuse during their youth. These men spoke of early life exposure to parent-on-parent violence, childhood abuse and neglect, and familial substance abuse. Participants identify gambling during their youth as a means of escaping painful memories; to feel the rush and the “adrenalin,” feel “exhilarated” and “keep my mind off other worries I was having in my life”. These findings are consistent with preliminary research reporting that adverse childhood life events are associated with gambling behaviours among youth [27,28]. Thus, the men in this study gambled as youth to cope with painful and difficult circumstances.

Our results highlight the degree to which these men’s families were not always able to meet their economic, social, and emotional needs. Families may lack material resources and/or be internally dysfunctional (e.g., parental substance abuse, domestic violence). As a result, youth might be exposed to gambling activity within [35,49] and outside the family unit [50,51]. This, coupled with exposure to difficult family environments, contributes to increased risk of problem gambling. Mark, for example described the inability of social service agencies to keep his family intact and Nolan’s mother could not cope with his early criminality and asked him to leave, therefore he became homeless.

We found that exposure to street culture and the formation of street social networks contributed to early gambling. For example, Steve described how, in the absence of responsible adults, he had “no choice” but to join in the deviant behaviour of adults in his network (e.g., gambling and substance use). Similar to others, our findings suggest that homelessness may be a pathway into street cultures that expose youth to maladaptive behaviour (see Chamberlain and Johnson [52]). Our findings point to the need for additional research on the connection between lack of family cohesion/supervision, entry into homelessness or precarious housing and how youth become involved with adult peer groups that model and enable gambling behaviour.

For youth who are homeless, gambling functions as a mechanism to support oneself and to cope with trauma. Nolan used gambling to keep his “mind off worries” including housing issues. Elliot saw gambling as a skill-based activity to support himself while living on his own during high school. In the only other study of gambling among homeless youth, Dufour, Roy, Boivin, Boudreau and Robert [11] suggested that youth in precarious living situations may turn to gambling to support themselves when resources are scarce [11]. As Robinson [53] argues, inhabitation itself creates grief and trauma. The experience of being displaced or the experience of abusive past home environments creates incredible trauma that youth carry with them throughout their lives; some youth will turn to substance use to cope with this grief and trauma [53]. Others will potentially develop mental health concerns such as anxiety, depression [54] and PTSD [55] (see Edidin et al. [56] for a comprehensive review). Indeed, research suggests that mental health issues are correlated with homelessness [57,58,59]. While the men’s narratives did not describe formal diagnoses of mental illness as youth, they spoke about their struggle to cope with their experience of trauma (abuse, homelessness) in their early lives. Our findings suggest that youth experiencing homelessness may also turn to gambling to cope with adverse life circumstances and point to the importance of developing problem gambling prevention programs and interventions that emphasize effective coping strategies for youth who have faced adversity.

As noted in previous research, positive experiences with gambling in youth, like a big win, are risk factors for the development of problem gambling [23,25]. Many of the men in this study described an early big win in youth as a very memorable turning point in their gambling experience which was potentially more striking in the context of homelessness and poverty. Men also described that gambling was interrelated with substance use. Elliot described that gambling in youth provided “an avenue to obtain drugs”, and Nolan stated that gambling was “natural” when he was drinking. Fahad said: “I started gambling when I started drinking.” These findings are in line with those from a recent literature review of youth and adult studies demonstrating that gambling coexists with other addictions [60]. We also found this relationship extended to the broader sample of men in our study who struggled concurrently with substance use and gambling, potentially mutually reinforcing each other [61] .

There is research that concludes that early interventions may help to support families that are struggling with complex vulnerabilities [62,63,64]. Our research suggests that interventions should include targeted assistance for gambling. There are also educational gaps in helping families understand how gambling interacts with other complex health and social issues, particularly alcohol and drug use, and response to traumatic events. Interventions could assist parents by increasing awareness of the risks of exposure to gambling in youth, as it is recognized that parents’ attitudes and gambling behaviours have an important influence on adolescent behaviour [65].

Positive familial role models are an important contributor to youth resiliency [48] and can play a pivotal role in interventions for youth [66] yet homeless youth and youth living in foster care may lack a parental or familial role model. In these circumstances, it may be beneficial to engage social service workers, youth criminal justice workers, health care professionals and school staff as critical intervention points for screening and treating problem gambling. Providing education for these professionals on recognizing and providing support for problem gambling may be useful.

Ultimately, the present findings suggest that youth experiences with trauma, neglect, poverty, and abuse may contribute to early participation in gambling behaviour. As a result, we recommend that any interventions use a holistic trauma informed approach to support youth and families with complex needs [67].

### Limitations

This study was conducted with men in a single urban centre, as such their experiences may not be generalizable to women, but the findings may inform future studies with female participants reflecting on their youth. There is also the potential for recall bias as men relied on memory of childhood experiences. It is also difficult to unravel the influence of problem gambling in shaping the life trajectory of our participants, as they also experience other life difficulties (e.g., early poverty, homelessness, substance use, trauma). The findings do not allow us to establish the temporal order of gambling, homelessness and other adverse life events but do provide a picture of how complicated their lives were in youth. Other studies will need to explore this in more detail. Our findings suggest that for some youth the experience of vulnerable social environments may lead to development of problem gambling in youth and persistence of problematic gambling over the life course.

## 5. Conclusions

Problem gambling among youth in an important public health concern. Responsible messaging and an appreciation and awareness of the risk of problem gambling in the context of more vulnerable youth should be on the radar of public health agencies and should be part of responsible gambling messaging to youth, their families and social and human services providers. Participants reported surviving poverty when faced with early independence from the family unit. This theme echoes results from other research in this area that has explored the role of early life trauma and its association with problem and or pathological gambling. The men described that gambling became a way for them to escape from painful experiences and alleviate stress experienced during youth, and in part, helped them survive early independence from their family unit as a result of adversity (e.g., homelessness, and abuse) by helping them earn a living. Gambling was perceived as an activity to provide much needed funds to afford basic necessities, and in some cases, to fund a co-existing substance addiction. Gambling became a central coping strategy and a way to establish self-identity in the absence of healthy role models and adequate social supports. Gambling was an activity that contributed to the men’s sense of self as youth, a way to be good at something, or a way to fit in within their social networks. This paper begins to address one of the recommendations by Dufour, Roy, Boivin, Boudreau and Robert [11]), to explore the “characteristics of homeless youth at risk of developing gambling problems”. Future research should explore how experiences of gambling differ among younger and older youth. This study is one of the first studies to illustrate how gambling develops in the landscape of early life exposure to adversity such as family violence and homelessness. We also begin to unravel the connection between early exposure specifically to gambling and adult problem gambling. 

## Figures and Tables

**Table 1:** Experiences of adversity in youth as reported by case study participants.

Pseudonym	Childhood Abuse/Neglect	Substance Use before Age 18	Homelessness before Age 18	Poverty
Mark	x	x	x	x
Steve	x	x	x	x
Fahad	x	x	x	x
Elliot		x	x	
Nolan	x	x	x	

**Table 2:** Reasons for gambling in youth as reported by case study participants.

Pseudonym	Earn Money for Basic Needs	Earn Money to Buy Drugs or Alcohol	Escape Painful Memories	Adrenalin High or Rush	Social Reinforcement and/or Perceived Skill	Early Big Win Reported
Mark			x	x		x
Steve	x	x			x	
Fahad		x		x	x	x
Elliot	x	x			x	x
Nolan		x	x	x		x

## References

[1] Blinn-Pike L., Worthy S.L., Jonkman J.N. (2010). Adolescent gambling: A review of an emerging field of research. J. Adolesc. Health.

[2] Messerlian C., Derevensky J., Gupta R. (2005). Youth gambling problems: A public health perspective. Health Promot. Int..

[3] Volberg R.A., Gupta R., Griffiths M.D., Olason D.T., Delfabbro P. (2010). An international perspective on youth gambling prevalence studies. Int. J. Adolesc. Med. Health.

[4] Derevensky J.L., Shek D.T.L., Merrik J. (2011). Youth Gambling: The Hidden Addiction.

[5] Hardoon K.K., Derevensky J.L. (2002). Child and adolescent gambling behavior: Current knowledge. Clin. Child Psychol. Psychiatry.

[6] Derevensky J.L., Gupta R. (2000). Prevalence estimates of adolescent gambling: A comparison of the SOGS-RA, DSM-IV-J, and the GA 20 questions. J. Gambl. Stud..

[7] National Research Council Commission on Behavioral Social Sciences Education (1999). Pathological Gambling: A Critical Review.

[8] Dickson L., Derevensky J.L. (2006). Equipping school psychologists to address another risky behavior: The case for understanding youth problem gambling. Can. J. Sch. Psychol..

[9] Matheson F.I., Devotta K., Wendaferew A., Pedersen C. (2014). Prevalence of gambling problems among the clients of a Toronto homeless shelter. J. Gambl. Stud..

[10] Sharman S., Dreyer J., Aitken M., Clark L., Bowden-Jones H. (2014). Rates of problematic gambling in a British homeless sample: A preliminary study. J. Gambl. Stud..

[11] Dufour M., Roy E., Boivin J., Boudreau J., Robert M. (2014). Correlates of at-risk gambling behaviors of homeless youth. J. Addict. Res. Ther..

[12] Dickson L., Derevensky J.L., Gupta R. (2008). Youth gambling problems: Examining risk and protective factors. Int. Gambl. Stud..

[13] Lynch W.J., Maciejewski P.K., Potenza M.N. (2004). Psychiatric correlates of gambling in adolescents and young adults grouped by age at gambling onset. Arch. Gen. Psychiatry.

[14] Barnes G.M., Welte J.W., Hoffman J.H., Tidwell M.C. (2011). The co-occurrence of gambling with substance use and conduct disorder among youth in the United States. Am. J. Addict..

[15] Potenza M.N., Wareham J.D., Steinberg M.A., Rugle L., Cavallo D.A., Krishnan-Sarin S., Desai R.A. (2011). Correlates of at-risk/problem internet gambling in adolescents. J. Am. Acad. Child Adolesc. Psychiatry.

[16] Shead N.W., Derevensky J.L., Gupta R. (2010). Risk and protective factors associated with youth problem gambling. Int. J. Adolesc. Med. Health.

[17] Hardoon K.K. (2004). An examination of psychosocial variables involved in adolescent gambling and high-risk behaviors. Diss. Abstr. Int. Sect. Humanit. Soc. Sci..

[18] Kristiansen S.G., Jensen S.M. (2014). Prevalence and correlates of problematic gambling among Danish adolescents. Int. J. Soc. Welf..

[19] Barnes G.M., Welte J.W., Hoffman J.H., Tidwell M.C. (2010). Comparisons of gambling and alcohol use among college students and noncollege young people in the United States. J. Am. Coll. Health.

[20] Desai R.A., Maciejewski P.K., Pantalon M.V., Potenza M.N. (2005). Gender differences in adolescent gambling. Ann. Clin. Psychiatry.

[21] Chalmers H., Willoughby T. (2006). Do predictors of gambling involvement differ across male and female adolescents?. J. Gambl. Stud..

[22] Barnes G.M., Welte J.W., Hoffman J.H., Dintcheff B.A. (2005). Shared predictors of youthful gambling, substance use, and delinquency. Psychol. Addict. Behav..

[23] Delfabbro P., King D., Griffiths M.D. (2014). From adolescent to adult gambling: An analysis of longitudinal gambling patterns in South Australia. J. Gambl. Stud..

[24] Shaffer H.J., Hall M.N. (2001). Updating and refining prevalence estimates of disordered gambling behaviour in the United States and Canada. Can. J. Public Health.

[25] Mentzoni R.A., Laberg J.C., Brunborg G.S., Molde H., Pallesen S. (2012). Tempo in electronic gaming machines affects behavior among at-risk gamblers. J. Behav. Addict..

[26] Fabiansson C. (2006). Recreational gambling: Young people’s gambling participation in rural Australia. J. Youth Stud..

[27] Storr C.L., Lee G.P., Derevensky J.L., Ialongo N.S., Martins S.S. (2012). Gambling and adverse life events among urban adolescents. J. Gambl. Stud..

[28] Lee G.P., Storr C.L., Ialongo N.S., Martins S.S. (2012). Association between adverse life events and addictive behaviors among male and female adolescents. Am. J. Addict..

[29] Scherrer J.F., Xian H., Kapp J.M.K., Waterman B., Shah K.R., Volberg R., Eisen S.A. (2007). Association between exposure to childhood and lifetime traumatic events and lifetime pathological gambling in a twin cohort. J. Nerv. Ment. Dis..

[30] Lee B., Solowoniuk J., Fong M. (2007). “I was independent since I was born”: Pre-immigration traumatic experiences and pathological gambling in four Chinese Canadians. Int. J. Migr. Health Soc. Care.

[31] Sharma A., Sacco P. (2015). Adverse childhood experiences and gambling: Results from a national survey. J. Soc. Work Pract. Addict..

[32] Hodgins D.C., Schopflocher D.P., el-Guebaly N., Casey D.M., Smith G.J., Williams R.J., Wood R.T. (2010). The association between childhood maltreatment and gambling problems in a community sample of adult men and women. Psychol. Addict. Behav..

[33] Saugeres L., Thomas A., Moore S. (2014). “It wasn’t a very encouraging environment”: Influence of early family experiences on problem and at-risk gamblers in Victoria, Australia. Int. Gambl. Stud..

[34] Shead N.W., Walsh K., Taylor A., Derevensky J.L., Gupta R. (2011). Youth gambling prevention: Can public service announcements featuring celebrity spokespersons be effective?. Int. J. Ment. Health Addict..

[35] Reith G., Dobbie F. (2011). Beginning gambling: The role of social networks and environment. Addict. Res. Theory.

[36] Griffiths M., Linsey A. (2006). Adolescent gambling: Still a cause for concern?. Educ. Health.

[37] Hodgins D.C. (2004). Using the NORC DSM screen for gambling problems as an outcome measure for pathological gambling: Psychometric evaluation. Addict Behav..

[38] Toce-Gerstein M., Gerstein D.R., Volberg R.A. (2009). The NODS-CLiP: A rapid screen for adult pathological and problem gambling. J. Gambl. Stud..

[39] American Psychiatric Association (1994). Diagnostic and Statistical Manual of Mental Disorders.

[40] Morse J.M. (2000). Determining sample size. Qual. Health Res..

[41] Devotta K., Woodhall-Melnik J., Pedersen C., Wendaferew A., Dowbor T.P., Guilcher S.J., Hamilton-Wright S., Ferentzy P., Hwang S.W. (2016). Enriching qualitative research by engaging peer interviewers: A case study. Qual. Res..

[42] Kessler R.C., Andrews G., Colpe L.J., Hiripi E., Mroczek D.K., Normand S.-L., Walters E.E., Zaslavsky A.M. (2002). Short screening scales to monitor population prevalences and trends in non-specific psychological distress. Psychol. Med..

[43] Humeniuk R., Ali R., ASSIST Phase II Study Group (2006). Validation of the Alcohol, Smoking and Substance Involvement Screening Test (ASSIST) and Pilot Brief Intervention (Electronic Resource): A Technical Report of Phase II Findings of the Who ASSIST Project.

[44] Glaser B.G., Strauss A.L. (1967). The Discovery of Grounded Theory: Strategies for Qualitative Research.

[45] Burnard P. (1991). A method of analysing interview transcripts in qualitative research. Nurse Educ. Today.

[46] Froonjian J., Garnett J.L. (2013). Reaching the hard to reach: Drawing lessons from research and practice. Int. J. Public Admin..

[47] Anthony E.K., Robbins D.E. (2013). A latent class analysis of resilient development among early adolescents living in public housing. Child. Youth Serv. Rev..

[48] Tozer K., Tzemis D., Amlani A., Coser L., Taylor D., Van Borek N., Saewyc E., Buxton J.A. (2015). Reorienting risk to resilience: Street-involved youth perspectives on preventing the transition to injection drug use. BMC Public Health.

[49] Orford J., Sproston K., Erens B., White C., Mitchell L. (2003). Gambling and Problem Gambling in Britain.

[50] Delfabbro P., Thrupp L. (2003). The social determinants of youth gambling in South Australian adolescents. J. Adolesc..

[51] Donati M.A., Chiesi F., Primi C. (2013). A model to explain at-risk/problem gambling among male and female adolescents: Gender similarities and differences. J. Adolesc..

[52] Chamberlain C., Johnson G. (2013). Pathways into adult homelessness. J. Sociol..

[53] Robinson C. (2005). Grieving home. Soc. Cult. Geogr..

[54] Yu M., North C.S., LaVesser P.D., Osborne V.A., Spitznagel E.L. (2008). A comparison study of psychiatric and behavior disorders and cognitive ability among homeless and housed children. Commun. Ment. Health J..

[55] Busen N.H., Engebretson J.C. (2008). Facilitating risk reduction among homeless and street-involved youth. J. Am. Acad. Nurse Pract..

[56] Edidin J.P., Ganim Z., Hunter S.J., Karnik N.S. (2012). The mental and physical health of homeless youth: A literature review. Child Psychiatry Hum. Dev..

[57] Fazel S., Khosla V., Doll H., Geddes J. (2008). The prevalence of mental disorders among the homeless in Western countries: Systematic review and meta-regression analysis. PLoS Med..

[58] Frankish C.J., Hwang S.W., Quantz D. (2005). Homelessness and health in Canada: Research lessons and priorities. Can. J. Public Health.

[59] Nower L., Eyrich-Garg K.M., Pollio D.E., North C.S. (2015). Problem gambling and homelessness: Results from an epidemiologic study. J. Gambl. Stud..

[60] Ferentzy P., Wayne Skinner W., Matheson F.I. (2013). Illicit drug use and problem gambling. ISRN Addict..

[61] Matheson F.I., Woodhall-Melnik J., Hamilton-Wright S., Guilcher S.J.T., Devotta K., Poremski D., Mohan A., Ferentzy P., Skinner W., Hwang S.W. (2015). Findings from the Problem Gambling and Housing Instability Study.

[62] Fantuzzo J., LeBoeuf W., Brumley B., Perlman S. (2013). A population-based inquiry of homeless episode characteristics and early educational well-being. Child. Youth Serv. Rev..

[63] Bassuk E.L., Richard M.K., Tsertsvadze A. (2015). The prevalence of mental illness in homeless children: A systematic review and meta-analysis. J. Am. Acad. Child Adolesc. Psychiatry.

[64] Haskett M.E., Armstrong J.M., Tisdale J. (2016). Developmental status and social–emotional functioning of young children experiencing homelessness. ECEJ.

[65] McComb J.L., Sabiston C.M. (2010). Family influences on adolescent gambling behavior: A review of the literature. J. Gambl. Stud..

[66] Mayock P., Corr M.L., O’Sullivan E. (2011). Homeless young people, families and change: Family support as a facilitator to exiting homelessness. Child Fam. Soc. Work.

[67] Jean Tweed Centre (2013). Trauma Matters: Guidelines for Trauma-Informed Practices in Women’s Substance Use Services.

